# Honing in on disease etiology

**DOI:** 10.4102/ajlm.v6i1.679

**Published:** 2017-12-08

**Authors:** Iruka N. Okeke

**Affiliations:** 1 Department of Pharmaceutical Microbiology, University of Ibadan, Ibadan, Nigeria

What are the causes of disease? How best can laboratories detect them? And what can we do with the knowledge that laboratories generate to improve disease prevention, containment and treatment? These are questions that fall within the *African Journal of Laboratory Medicine*’s scope and for which some poignant answers were supplied by articles in this volume.

Our cover features a photomicrograph of *Cylindrocarpon lichenicola* from Irek and colleagues’ report of a rare but debilitating case of keratomycosis^1^. A skilled diagnostician can only confirm this infection with access to mycology laboratory resources, which are uncommon on the African continent. This case report of a soil-acquired infection in a farmer points to the need for diagnostic laboratory vigilance, as well as a need to advocate for broader fungal diagnostics and treatments for African patients.

For more common conditions, access to laboratory testing is critical for public health, as well as patient care. Inadequate access to testing stands in the way of HIV care for roughly half of Africans infected with the virus. Inadequate access was also a significant factor in the challenge to contain the recent Ebola outbreak in West Africa. Articles in this issue report on how the introduction of testing interventions positively impacted disease care on the continent for both life-threatening infections. In one example of how diagnostic test development and deployment can overcome health worker shortages, Kaindjee-Tjituka et al.^2^ found that introduction of point-of-care CD4 testing in Namibia allows testing to be performed by nurses and lay community counsellors. Thus, patients received test results and counselling on the same day and in some cases antiretroviral therapy was speedily initiated. Louis and co-workers^3^ detail the parallel rapid diagnostic testing for malaria and Ebola for febrile patients and corpses in Forécariah, west Guinea, in 2015. Healthcare workers and Red Cross volunteers performed the tests, and their ability to care for patients and deliver healthcare safely was positively impacted. However, they faced some challenges such as: patients equating Ebola testing with being diseased and the limited sensitivity of the Ebola rapid diagnostic test, which meant that corpses that tested negative were still buried according to safe haemorrhagic fever burial practices.

One of the criteria for promoting tests that are evaluated at the point of care is ease of interpretation, which allows trained lay people as well as health professionals to administer testing and respond appropriately to results. However, not all test encounters are this straightforward. Primary healthcare physicians use a wide variety of tests to inform their diagnoses, and 17% of those surveyed by Vanker and Faull^4^ in South Africa reported that they were unsure of how to interpret or use test results. Vanker and Faull state that this percentage is roughly double that reported from the United States, and their research points to the need for better physician education and support and clear test reporting, particularly in those areas that pinpoint disease etiology: microbiology, serology and discordant HIV tests.

Good testing is essential for optimal patient management, particularly in resource-limited settings, and also informs surveillance. As Uwimana et al.^5^ report, under 30% of all leprosy cases in Rwanda within the 17-year period between 1995 and 2011 were verified by laboratory results. The good news from that article is that leprosy detection rates are dropping – a testament to improved surveillance – and that laboratory verification of cases is becoming more frequent. In 2010, roughly 70% of cases were laboratory-verified, whereas 10 years prior, under 5% of reported cases were supported by laboratory results.

Almost all of the articles in this issue emphasise the need for continued improvement in access to laboratory testing and in surveillance for infectious diseases across the continent. Thus, it is refreshing to learn from the guest editorial in this issue by Amukele^6^ that the Africa Centres for Disease Control has made a strong start. This is certainly an exciting time for me to have commenced my tenure as *African Journal of Laboratory Medicine*’s Editor-in-Chief.
